# Coordinated gene expression of Th17- and Treg-associated molecules correlated with resolution of the monophasic experimental autoimmune uveitis

**Published:** 2011-06-07

**Authors:** Xiuzhi Jia, Minghui Hu, Caihong Wang, Chunyu Wang, Fengyun Zhang, Qinglian Han, Ruibo Zhao, Qi Huang, Hongwei Xu, Huiping Yuan, Huan Ren

**Affiliations:** 1 Department of Immunology, Harbin Medical University, Harbin, China; 2 Infection and Immunity, Key Laboratory of Heilongjiang Province, Harbin, China; 3 Department of Pathology, Harbin Medical University, Harbin, China; 4 Department of Ophthalmology, Second Affiliated Hospital to Harbin Medical University, Harbin, China

## Abstract

**Purpose:**

To investigate the role of T-cell-mediated immune response in a monophasic experimental autoimmune uveitis (EAU).

**Methods:**

A monophasic EAU was induced in Lewis rats by immunization with interphotoreceptor retinoid-binding protein peptide. Optimized quantitative real-time RT–PCR was used for consecutive measurement of the relative expression of Th17-associated molecules, including interleukin 6 (*IL-6*), transforming growth factor-β (*TGF-β*), interleukin 23p19 (*IL-23p19*), interleukin 23p40 (*IL-23p40*), *CD4*, *CD8*, major histocompatibility complex I (*MHC I*), major histocompatibility complex II (*MHC II*), interleukin 17 (*IL-17*), interleukin 17F (*IL-17F*), interleukin 17 receptor A (*IL-17RA*), retinoic acid-related orphan receptor γt (*RORγt*) and Chemokine receptor 6 (*CCR6*), in addition to Treg-related forkhead box P3 (*Foxp3*), C-X-C chemokine receptor type 5 (*CXCR5*), and cluster of differentiation 25 (*CD25*) at the initiation, effector, and resolution phases of EAU and compared with those at 14 days post-immunization of control animals. Immunohistochemisty was used to examine IL-17 expression in retinas. Glial fibrillary acidic protein retinal astrocytes, Neuronal class III β-Tubulin(Tuj1^+^)retinal ganglion cells, and infiltrating CD11b^+^ microglia were analyzed by fluorescent microscopy in a kinetic manner.

**Results:**

Our results indicated well organized T-cell activity, measured by relative expression of multiple T-cell-related factors at the mRNA level, synchronized with the initiation of autoimmune inflammation, and thereafter resolution of the monophasic EAU. Immune balance was achieved several times through coordinated expression of Th17- and Treg-related factors. The expression pattern of these factors and results from immunochemistry with an IL-17 antibody indicated that there may be intensive crosstalk between infiltrating immune cells and the resident neural cells, which were significantly activated during the course of disease.

**Conclusions:**

T-cell-mediated immune response played a positive role in resolution of the monophasic EAU.

## Introduction

Experimental autoimmune uveitis (EAU) is an organ-specific, T-cell-mediated, autoimmune disease that targets the neural retina and related tissues [1]. The pathology of EAU resembles human uveitic diseases of a recognized autoimmune nature in which patients present with immunological responses to retinal antigens [1,2]. Immunization with the immunodominant 1177–1191 peptide (R16) of interphotoreceptor retinoid-binding protein (IRBP) results in a monophasic EAU in susceptible Lewis rats in which intraocular inflammation appears around day 8 after immunization, lasts for approximately 2 weeks, and then spontaneously resolves [3]. In contrast, recurrent uveitis that can be induced by adoptive transfer of antigen-specific lymphocytes is characterized by recurring attacks of CD4+ T cells targeting intraocular structures, particularly the retina. As the retina assembles as part of the central nervous system (CNS), affected areas with destructive architecture are unable to reorganize and therefore remain functionally compromised [1,3]. Over the years, efforts have focused on understanding the mechanisms involved in the natural resolution of monophasic uveitis and identifying optimal therapeutic strategies for human recurrent or chronic uveitis.

The nature of monophasic EAU to spontaneously resolve after tremendous ocular inflammation suggests that the involved T-cell autoimmunity could be a beneficial physiologic response evoked to defend, repair, and maintain. At the effector stage of EAU or other autoimmune diseases, high levels of interferon-γ (IFN-γ) or interleukin 17 (IL-17), produced by T-helper type 1 (Th1) or Th17 cells, were believed to be critically pathogenic [2,4,5]. However, evidence indicates that IFN-γ affects glutamate uptake by resident astrocytes in mice subjected to optic nerve injury [6,7]. The dual nature of IL-17A to be either pathogenic or protective has been reported, and its specific function may depend on the stage of different autoimmune diseases [5,8,9]. On the other hand, during autoimmune inflammation in the CNS, astrocytes and microglia that encounter adaptive immunity could be activated, become capable of presenting antigens and engaging in a dialog with T cells, and perform protective activities against local threats [10]. A recent report indicated that astrocytes play an important physiologic role in CNS homeostasis and could serve as a target of Th17 and IL-17 [11]. Other relevant studies have suggested that neuron- [12] or astrocyte- [13] induced regulatory T cells (Treg) may represent an important mechanism for self-limiting excessive inflammation in the brain. Further evidence has shown that retinal ganglion cells (RGCs) exposed to a glutamate insult or suffering the secondary consequences of an optic nerve crush injury could be protected by vaccination with IRBP in both susceptible Lewis rats and Fisher rats resistant to EAU [14].

Heterogenity and plasticity of effector/regulatory T-cell subsets and function of a given T-cell lineage involved in autoimmune inflammation are currently being debated. Immunosuppressive Treg and pro-inflammatory Th17 cells functionally antagonize each other. However, as their differentiation programs are reciprocally inter-related, recent data has revealed that they may support each other in differentiation and expansion. For example, in the presence of interleukin 6 (IL-6) or interleukin 1β (IL-1β), transforming growth factor-β (TGF-β) produced by Treg may promote the generation of Th17 cells from naïve CD4 cells [15,16]. Plasticity of Treg was recently demonstrated. Treg are reported to lose their suppressive function and be reprogrammed to the Th17 phenotype in the presence of TGF-β and IL-6 [16-18]. Significant numbers of CD4 T cells co-expressing forkhead box P3 (Foxp3) and retinoic acid-related orphan receptor γt (RORγt) transcriptional factors have been found in human peripheral tissues; these cells produced IL-17 and powerfully inhibited the proliferation of CD4 responder cells [19]. Thus, the ability to suppress responder cells and at the same time generate IL-17 may render these cells with a twin role in antimicrobial defense, while controlling autoimmunity and inflammation. These and other reports indicate that the interplay and interconnection between Th17 and Treg are much more complex than generally appreciated [20,21].

With the use of large-scale analysis approaches, it should be possible to extract more detailed information out of these complex biologic systems and understand the complex biology and mechanisms by which inappropriate T-cell activity results in chronic pathology. In this study by measuring the consecutive expression of multiple Th17- and Treg-related molecules locally at the mRNA level and systematically in the monophasic EAU with Lewis rats, we showed how kinetics of effector/regulatory T-cell activity synchronized the initiation and resolution of autoimmune inflammation in vivo. In addition, the expression pattern of these inter-related molecules and other related data support intensive crosstalk between the infiltrating immune cells and resident neural cells in the course of disease.

## Methods

### Animals

Female Lewis rats (160–180 g, 5–6 weeks) of specified pathogen-free grade were purchased from Peking Vital River Laboratory Animal Ltd. (Beijing, China). All rats were fed and maintained in specified pathogen-free conditions according to the guidelines of Care and Use of Laboratory Animals published by the China National Institute of Health. All experimental procedures adhered to the Association for Research in Vision and Ophthalmology Statement for the use of animals in ophthalmic and vision research.

### Induction of experimental autoimmune uveitis

Peptide R16 of bovine IRBP (residues 1177–1191, sequence ADGSSWEGVGVVPDV) was synthesized by solid-phase techniques and puriﬁed by HPLC (Chinese Peptide Co., Hangzhou, China). The peptide was prepared by emulsification of 50 µg IRBP R16 peptide in Freund's Complete Adjuvant CFA (Sigma-Aldrich, St. Louis, MO) containing 2.5 mg/ml of mycobacterium tuberculosis H37Ra in a total volume of 0.1 ml. Rats were divided into two groups; the EAU group received an injection of 0.1 ml peptide antigen in each of the footpads and the CFA control received an equal volume of injection at the same locations with PBS emulsified with CFA. Concurrently, both groups of animals were intraperitoneally injected with 1 μg of pertussis toxin (Sigma-Aldrich) [3] in a volume of 0.1 ml for more accurate onset of EAU. Animals were monitored daily, and specified tissues were collected for further studies. Three batches of immunization, each including 35 Lewis rats, were used for EAU induction, while the respective control of 15 animals was also prepared.

### Clinical and histopathological assessment

All animals were monitored daily. Clinical signs were scored by slit-lamp biomicroscopy according to the following criteria [3]: 0, the eye is translucent and reflects light; 0.5, dilated blood vessels in the iris; 1.0, engorged blood vessels in the iris, abnormal pupil contraction; 2.0, hazy anterior chamber, decreased red reflex; 3.0, moderately opaque anterior chamber but pupil still visible, dull red reflex; 4.0, opaque anterior chamber and obscured pupil, red reflex absent, proptosis. Clinical scores were analyzed using a nonparametric Mann–Whitney U test; p<0.05 was regarded as statistically significant.

The degree of eye inflammation was also confirmed by histopathological analysis. Briefly, the entire eye ball of euthanized animals was freshly dissected and fixed in 4% formaldehyde for 24 h, before the corneas were cut and lens carefully removed. Fixed eyes were routinely embedded in paraffin blocks, and 2-μm sections were stained with hematoxylin and eosin (Zhongshan Goldenbridge, Guangzhou, China). The presence of disease was evaluated blindly by examining six sections cut through the pupillary–optic nerve plane at different levels for each eye. Severity of EAU was scored on a scale of 0 (no disease) to 4 (maximum disease) [3]: 0, no disease, normal retinal architecture; 0.5, mild inflammatory cell infiltration of the retina with or without photoreceptor damage; 1.0, mild inflammation and/or photoreceptor outer segment damage; 2.0, mild to moderate inflammation and/or lesion extending to the outer nuclear layer; 3.0, moderate to marked inflammation and/or lesion extending to the inner nuclear layer; 4.0, severe inflammation and/or full-thickness retinal damage.

### Quantitative real-time RT–PCR

To avoid variations among different batches of immunizations and individual rats in the same batch, two rats of the same immunization batch at each selected time point (with close clinical scores on their eyes) were used for quantitative real-time RT–PCR analysis. Tissue samples from two rats of the CFA group at 14 days post immunization (dpi) were used as controls. Data from the third batch of immunization are shown in Table 1, Table 2, and Table 3. Consistent results were obtained among batches of immunization. Briefly, freshly isolated retinas, draining lymph nodes, and spleens from selected EAU and control animals were rapidly embedded in RNA*later* (Sigma-Aldrich) and stored at –20 °C. Total RNA was extracted from these tissues with Trizol reagent (Invitrogen, Shanghai, China), and 1 µg of RNA was reverse transcribed using High-Capacity cDNA Reverse Transcription kits (Applied Biosystems, Foster City, CA). The cDNA solution was mixed into one bulk solution for each kind of tissue from selected EAU or control rats at each time point, and the relative expression of Th17- or Treg-associated molecules at the mRNA level was analyzed.

**Table 1:** Relative expression (fold change of control) of Th17-associated and Treg-related factors at the mRNA level from draining lymph nodes during monophasic EAU with Lewis rats.

**Genes**	**con**	**7 d**	**14 d**	**21 d**	**28 d**	**35 d**
**Th17-related**						
IL-6	1.00	**7.67**	1.76	**2.73**	1.57	1.25
TGF-β	1.00	0.61	0.85	1.41	1.21	1.31
IL-23p19	1.00	0.63	1.00	0.65	0.80	0.81
IL-23 (−12)p40	1.00	*0.26*	0.82	0.88	1.16	1.28
IL-17	1.00	**42.63**	**20.22**	1.51	**3.21**	**1.94**
IL-17F	1.00	**15.77**	**2.42**	0.98	0.91	0.84
IL-17R	1.00	*0.28*	*0.30*	*0.02*	*0.09*	*0.08*
CD4	1.00	0.63	0.88	1.23	1.05	1.10
MHC II	1.00	1.74	**2.48**	0.88	1.25	1.09
CD8	1.00	0.91	1.04	1.22	1.10	1.58
MHC I	1.00	1.06	1.35	0.67	0.84	0.90
RORγt	1.00	1.76	1.64	1.74	1.67	1.88
CCR6	1.00	0.74	0.85	1.39	1.09	1.35
**Treg-related**						
Foxp3	1.00	*0.44*	0.68	0.77	0.81	0.93
CXCR5	1.00	1.97	**2.72**	0.92	1.12	1.22
CD25	1.00	0.72	0.81	1.12	0.89	1.25

Notes: con*, tissue counterparts were obtained from control rats, relative expression of the gene of interest was measured by the same method with the EAU rats and set as 1.00, respectively. Genes showing at least a twofold change were recognized as being significantly altered. Significantly increased expression was labeled in bold and decreased in italic.

**Table 2:** Relative expression (fold change of control) of Th17-associated and Treg-related factors at the mRNA level from the spleen during monophasic EAU with Lewis rats.

**Genes**	**con**	**7 d**	**14 d**	**21 d**	**28 d**	**35 d**
**Th17 related**						
IL-6	1.00	0.69	0.90	0.78	1.06	1.09
TGF-β	1.00	0.90	0.92	0.67	0.89	0.91
IL-23p19	1.00	0.97	1.11	0.90	1.27	1.24
IL-23 (−12) p40	1.00	0.60	0.76	0.85	0.75	0.86
IL-17	ND^$^	ND^$^	ND^$^	ND^$^	ND^$^	ND^$^
IL-17F	ND^$^	ND^$^	ND^$^	ND^$^	ND^$^	ND^$^
IL-17R	1.00	1.24	0.97	0.90	0.82	1.10
CD4	1.00	0.88	0.99	0.87	1.01	0.91
MHC II	1.00	0.79	0.74	0.72	0.84	0.73
CD8	1.00	1.54	0.56	0.53	0.71	0.65
MHC I	1.00	0.91	0.81	0.84	0.99	0.91
RORγt	1.00	1.20	1.83	1.07	1.15	1.20
CCR6	1.00	0.63	0.71	0.68	0.85	0.80
**Treg-related**						
Foxp3	1.00	0.90	0.82	0.70	0.87	0.82
CXCR5	1.00	1.04	0.99	0.90	0.94	1.17
CD25	1.00	0.95	0.97	0.80	0.89	0.81

Notes: con*, tissue counterparts were obtained from control Lewis rats, relative expression of the gene of interest was measured by the same method with the EAU rats and set as 1.00, respectively. ND^$^, the expression was too low to be detected. Genes showing at least a twofold change were recognized as being significantly altered.

**Table 3:** Relative expressions of Th17- and Treg- related factors at the mRNA level from retina during monophasic EAU with Lewis rats and standard Pearson correlation analysis.

**Genes**	**Relative expression as fold change**						**Correlation with**	**p**
** **	con*	**7 d**	**14 d**	**21 d**	**28 d**	**35 d**	**IL-17 (r)**	**(2-tailed)**
**Initiating factors**								
IL-6	1.00	1.62	**33.01**	**3.30**	**2.86**	**6.92**	0.882*	0.020
TGF-β	1.00	1.74	**12.47**	**4.44**	**4.10**	**5.53**	0.907*	0.013
**Expansion factors**								
IL-23p19	1.00	1.34	1.10	1.15	0.85	1.80	−0.035	0.947
IL-23 (−12) p40	1.00	*0.35*	**278.20**	**18.27**	**42.71**	**296.45**	0.954**	0.003
**Signature cytokines and receptor**								
IL-17	1.00	*0.10*	**1573.6**	**15.05**	**49.24**	**80.54**	—	—
IL-17F	1.00	0.99	**32.22**	0.89	1.49	1.47	0.741	0.092
IL-17R	1.00	1.85	**19.63**	**4.07**	**5.25**	**3.49**	0.874*	0.023
**Antigen presentation- associated factors**								
CD4	1.00	0.73	**36.13**	NA^#^	**8.47**	**4.05**	0.959**	0.010
MHC II	1.00	**7.31**	**5202.54**	**76.47**	**624.55**	**993.70**	0.906*	0.013
CD8	1.00	*0.22*	**9.58**	NA^#^	0.83	1.07	0.869	0.056
MHC I	1.00	0.95	**8.63**	0.66	1.60	**3.46**	0.790	0.061
**Lineage markers**								
RORγt	1.00	1.10	**6.02**	0.70	0.92	**3.09**	0.679	0.138
CCR6	1.00	1.41	**62.47**	**3.05**	**29.89**	**22.65**	0.911*	0.012
**Treg-related**								
Foxp3	1.00	0.86	**10.59**	0.84	1.59	**4.63**	0.839*	0.037
CXCR5	1.00	1.18	**8.08**	1.54	**2.58**	**3.51**	0.948*	0.014
CD25	1.00	1.15	**146.02**	NA#	**18.72**	**5.14**	0.915*	0.029

Notes: The time series data are representative for experiments of three batches of immunization for inducing EAU model in Lewis rats as shown in Figure 1. con*, retina were obtained from CFA control rats at 14 dpi, the relative expression of each gene examined was set as 1.00, respectively; NA^#^, the data were not available. Genes showing at least a twofold change were recognized as being significantly altered. Significantly increased expression was labeled in bold and decreased in italic. Standard Pearson analysis was performed between the time series expressions of one of the examined molecules and that of IL-17. *Correlation is significant at the 0.05 level; **Correlation is significant at the 0.01 level (2-tailed).

The Vandesompele et al. [22] method was adopted for the selection of housekeeping genes as internal genomic controls. Five housekeeping genes, including heat shock protein 90kDa alpha class B member 1 (Hsp90ab1), ribosomal RNA 18s (18S), lactate dehydrogenase A (Ldha), arginine-glycine-aspartic acid (RGD), and ribosomal protein 13a (Rp113a) were tested on selected tissues (retinas, lymph nodes, and spleen) from control rats at 14 dpi for relevant gene stability. Four genes (*18s*, *Ldha*, *Hsp90ab1*, *Rp113a*) were used for normalization of the spleen and lymphoid node samples and three (*Ldha*, *RGD*, *Rp113a*) were used for the retina samples. Sequences of specific primers (Sangon Ltd., Shanghai, China) for the examined molecules are available in Appendix 1

**Figure 1:**
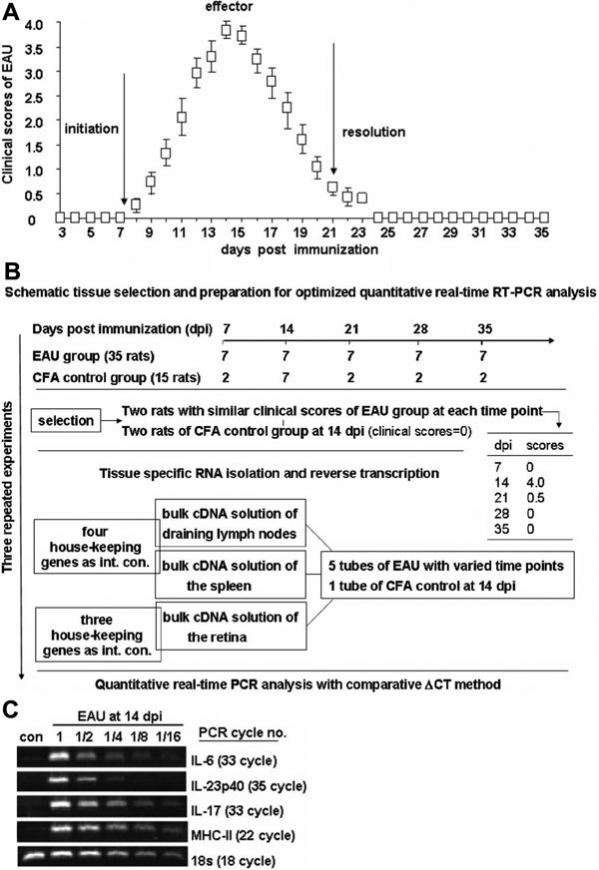
Clinical score of the monophasic EAU and methodology of real-time PCR analysis. **A**: Clinical scores of the monophasic experimental autoimmune uveitis (EAU). Disease severity was observed daily by slit-lamp microscopy and graded as described in the Methods section. Based on the clinical course, monophasic EAU was divided into three stages: an initiation phase from the day of immunization to 7 dpi; an effector phase beginning from 7 to 21 dpi, with the peak inflammation obtained at 14 dpi; and a phase of resolution starting from 21 dpi. Data are represented as the mean±standard deviation. **B**: Schematic tissue selection and preparation for optimized quantitative real-time RT–PCR analysis was shown. **C**: Validation of the results of quantitative real-time PCR by traditional PCR. Traditional PCR using the series diluted cDNA of EAU retina at 14 dpi and undiluted cDNA from the CFA control was performed, and the relative expression of the selected factors was compared with the results from quantitative real-time PCR. In line with the most significant 5,202 fold upregulation at EAU 14 dpi on MHC-II expression by real-time PCR analysis (Table 3), its relative expression was the most abundant as compared to that of other individually examined factors, e.g., *IL-17*, *IL-23p40*, and *IL-6*, by traditional PCR measurement. A visible PCR band on electrophoresis by using as little as 1/16 dilution of the original cDNA was observed in the series PCR for the expression of *MHC-II* with the lowest 22 PCR cycles compared to that of other sets of relevant PCR data, e.g., *IL-17* with 33 cycles. Data represent three repeated experiments.

The real-time PCR reaction was set up in a 15-µl volume using 2× FastStart Universal SYBR Green Master Mix (Roche Ltd., Basel, Switzerland) in which 0.375 μM of each primer and 0.5 μl cDNA were used. The PCR reaction was performed using an ABI STEPONE real-time PCR System (Applied Biosystems) with an initial denaturation of 95 °C for 10 min, followed by 40 cycles of 95 °C for 15 s and 60 °C for 1 min. Relative gene expression levels were quantified using the comparative ΔC_T_ method. This method normalized C_T_ values of the detected gene to the average of that of the housekeeping genes and calculated the relative expression values as fold changes of the control, which was set at 1.00. Melting curve analyses and electrophoresis was performed to verify the specificity of the PCR products. Each experiment was performed in duplicate and repeated two to three times, and the standard deviation among repeated experiments were stringently less than 2% when analyzed by the Student *t* test (data not shown). Tissue selection and preparation for optimized quantitative real-time RT–PCR analysis is shown in Figure 1B.

### Immunohistochemistry

Paraffin blocks of the eye from both EAU and control animals were prepared as described above. Antigen retrieval was performed by microwave-heating and nonspecific protein-binding sites were blocked by 4% normal goat serum plus 1% bovine serum albumin (BSA) in PBS for 30 min. The 2-um sections were incubated with an antirat IL-17 rabbit polyclonal antibody (1:100; Santa Cruz Biotechnology Inc., Santa Cruz, CA). Parallel nonimmune rabbit IgG was used as a negative control. Biotinylated secondary antibody, avidin:biotinylated enzyme complex, and 3,3′-diaminobenzidine substrate were used as detecting reagents (Zhongshan Goldenbridge Biotechnology). The slides were counterstained with hematoxylin and finally mounted with mounting medium and analyzed.

### Direct and indirect immunofluorescent microcopy

The eye cups were obtained from freshly dissected eye balls by carefully cutting off the corneas and removing the vitreous and lens on ice. Optimal Cutting Temperature™ O.C.T embedding medium (Richard-Allan Scientific, Kalamazoo, MI) was immediately applied to fill the eye cup, which was then snap frozen in liquid nitrogen and stored at −80 °C until use. Ten-micrometer sections at the coronal plane of the eye cup were cut by a cryostat (Microm HM525, Walldorf, Germany).

For sections subjected to indirect immunofluorescent microscopy, the samples were fixed in 4% paraformaldehye and then blocked in 4% goat serum in PBS containing 1% BSA and 0.6% Triton X-100. Primary antibody incubation was performed in 1% goat serum and 0.1% BSA in PBS at 4 °C overnight. These antibodies included antirat Tuj-1 (neuron-specific class III beta-tubulin) mouse monoclonal antibody (1:250; Covance Inc., Princeton, NJ) and antirat CD11b mouse monoclonal antibody (1:200; Abcam Inc., Cambridge, MA). Sections were incubated with FITC-conjugated or Texas Red-conjugated goat antimouse secondary antibody (1:200; Becton Dickinson, Franklin Lakes, NJ) in PBS containing 1% normal goat serum and 0.1% BSA for 1 h. The samples were then incubated with 2 μg/ml 2-(4-amidinophenyl) −6-indolecarbamidine dihydrochloride (DAPI; Roche, Basel, Switzerland) for 10 min and the slides were mounted. For sections subjected to direct immunofluorescence, FITC-conjugated antibody against GFAP (1:30; Biosynthesis Biotechnology Co. Ltd., Peking, China) was applied, and DAPI was used to counterstain the cell nucleus. Images were taken using a fluorescence microscope (Nikon 80*i*; Nikon, Tokyo, Japan) with a cold CCD camera (Nikon DS-Ri1; Nikon), which was equipped with NIS-Elements AR 3.0 software (Nikon). Positive staining was evaluated and analyzed blindly by a medical pathologist.

### Statistical analysis

Statistical evaluations of EAU scores and repeated real-time PCR data obtained from the same sample were performed by the Student *t* test. Standard Pearson correlation analysis on the real-time PCR data was performed to indicate significant correlation between the time series data of any of the tested T-cell-related factors and that of IL-17 in retina within each of the three repeated experiments (Figure 1B). Two-tailed p values were calculated. The level of significance was set to p<0.05 for both the Student *t* test and Pearson correlation analysis. The Pearson correlation r value was also calculated and interpreted as follows: 0.0 to 0.2, very weak to negligible correlation; 0.2 to 0.4, weak, low correlation; 0.4 to 0.7, moderate correlation; 0.7 to 0.9, strong, high correlation; 0.9 to 1.0, very strong correlation.

## Results

### Evaluation of the monophasic EAU, real-time RT–PCR design, and data validation

**Figure 2:**
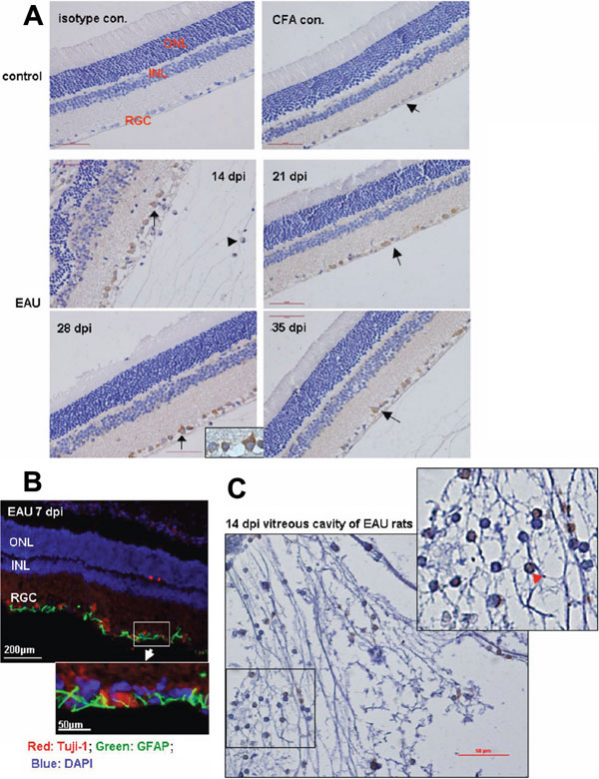
IL-17 was expressed by both resident neural cells and infiltrating lymphoid cells within the eyes of EAU animals. Immunohistochemistry with an IL-17-specific rabbit antirat antibody was employed to examine the intracellular staining on 7 (data not shown), 14, 21, 28, and 35 dpi of the retina and that of the CFA control at 14 dpi or vitreous cavity of the eye of EAU at 14 dpi. None-immune rabbit IgG was used as the antibody isotypic control applied on the retina of the CFA control. **A**: IL-17 expression at the retinas. No disease and normal retinal architecture was observed at both the isotype antibody control and CFA control. Disrupted retinal architecture and full-thickness retinal damage with intensive infiltrating cells were observed at 14 dpi, but the normal retina structure was gradually restored after 21 dpi. IL-17-positive staining was detected at the layer where retinal ganglion cells (RGC) and retinal astrocytes resided at the retina, with light staining at the retina of the CFA control and strong staining at 14, 21, 28, and 35 dpi of retinas of EAU rats (arrows). The network of neural processes also stained positively for IL-17. Closer observation on some of the positive-staining cells indicated that these cells morphologically resembled astrocytes (EAU 28 dpi, insert with the arrow). Furthermore, a certain amount of the infiltrating cells at 14 dpi of the EAU group also indicated positive staining (arrowhead). The size bar represents 50 μm (40×). **B**: GFAP+ retinal astrocytes and Tuj-1+RGC reside at the RGC layer of the retina. GFAP (Green); Tuj-1 (red), and DAPI (blue). **C**: Selected IL-17 expression at the vitreous cavity within the eyes of EAU 14 dpi. A significant amount of the infiltrating cells in the vitreous cavity within the eyes of EAU 14 dpi showed strong intracellular staining of IL-17 (arrowhead). The morphology of positive-staining cells infiltrating the retinas resembled cells of lymphoid origins. Data represent at least three repeated experiments. The size bar represents 50 μm (40×).

Each eye of both EAU and CFA control animals were observed daily after immunization until 35 dpi, and clinical scores were recorded (Figure 1A). Mild clinical signs, such as dilated or engorged blood vessels in the iris, were observed at 8–9 dpi, and the most severe intraocular inflammation was detected at 14 dpi, as evidenced by an opaque anterior chamber and obscured pupil. At 21 dpi, the ocular inflammation was greatly resolved, with only minor clinical signs, and no inflammatory signs were detected at 28 or 35 dpi. No indication of clinical EAU was observed in any of the CFA controls. All of these demonstrate an acute and monophasic disease course. For further detailed analysis, the course of EAU was separated into three phases, including an initiation phase from the day of immunization until 7 dpi; an effector phase from 7 to 21 dpi, in which the peak inflammation was obtained at 14 dpi; and a resolution phase beginning at 21 dpi (Figure 1A). Moreover, the results of clinical observation on each stage of disease were further confirmed by H&E histological analysis (data not shown) and immunochemistry with an IL-17 antibody (Figure 2A,C) in the retina and vitreous cavity within eyes from both groups of animals. Characteristic retinal folding, retina and vitreous infiltrates with EAU were observed in Figure 2A.

To examine the kinetics of related T-cell responses during the course of EAU systemically and locally, the relative abundance of mRNA for multiple T-cell-related molecules was measured by real-time PCR. Data shown (Table 1, Table 2, and Table 3) are from measurement of tissues collected from two of the representative EAU animals of one immunization and compared with that of the CFA controls at 14 dpi by the 2ΔC_T_ method [22] (Figure 1B). This set of experiments was repeated three times through three batches of immunization in Lewis rats, and similar real-time PCR data were obtained (data not shown). Because the collected draining lymph node, the spleen, and the retina are from the same two animals and thus systemically connected, we assumed that the expression levels or ratios of multiple Th17- or Treg-related factors among different tissues may be better comparable and reflect the inter-related linkage involved in the system’s biology. Furthermore, traditional PCR technology was also applied to verify some of the real-time PCR data (Figure 1C).

### Th17 contributed to the ocular inflammation at the effector phase of EAU in which Treg was also actively involved

Expression levels of T-cell-related molecules were analyzed by quantitative real-time PCR systemically with draining lymph nodes and the spleen and locally with the whole retinas from both EAU and CFA control rats at 7, 14, 21, 28, and 35 dpi. These molecules included the Th17 initiation cytokines (*IL-6* and *TGF-β*), the expansion cytokine *IL-23*, the Th17-signature cytokines and their relevant receptor (*IL-17*, *IL17F*, and *IL-17RA* as *IL-17R*), the Treg cell-surface markers (*CD4* and *CD25*), and other related cell-surface molecules, such as *CD8*, *MHC-I*, and *MHC-II*. In addition, key transcription factors for Th17 and Treg cells (*RORγt* and *Foxp3*) and chemokine receptors for both of the two T-cell subsets (*CCR6* and *CXCR5*) were also included (Table 1, Table 2, and Table 3).

Our kinetic data revealed that Th17 cells were initiated systemically within draining lymph nodes but not the spleen (Table 1 and Table 2). As in the spleen, the expression of most of those detected factors was not changed significantly with absolute fold changes of less than 2.00, as compared to the control (Table 2). Data from draining lymph nodes (Table 1) indicated that increased expression of *IL-6* and *IL-17* was observed at 7 and 14 dpi, both peaked at 7 dpi during the observation, with 7.67 and 42.62 fold upregulation, respectively, compared to the control. The relative expression of *IL-17F* also peaked at 7 dpi, with 15.77 fold upregulation, and remained elevated at 14 dpi with a 2.42 fold increase before returning to levels of the control at later phases. On the other hand, expression of *IL-17R* remained significantly decreased throughout the course of the experiment (Table 1). In addition, only slightly enhanced expression of the transcription factor *RORγt* and slightly reduced expression of the chemokine receptor *CCR6* at 7 and 14 dpi were observed over the entire course of observations. The expression of most other related factors, such as *CD4*, *CD8*, and *MHC-I*, did not change significantly at the time recorded, with the exception of *MHC-II*, which was elevated at 14 dpi by 2.48 fold compared to the control (Table 1). These sets of data indicated that, although Th17 cells might be induced and they produced high amounts of signature cytokines, including *IL-17* and *IL-17F*, at the initiation phase of EAU in the peripheral tissue, the draining lymph nodes may not be the target organ for these cells to mediate significant inflammation. Moreover, relative expression of the Treg cell “master regulator” *Foxp3* within the periphery was significantly downregulated at 7 dpi, whereas at 14 dpi the expression of the Treg-lineage-specific chemokine receptor CXCR5 was upregulated by 2.72 fold compared to the control (Table 1).

Data obtained from the retinas indicated that, with the most severe ocular inflammation being observed at 14 dpi with EAU rats (Figure 1 and Figure 2), it was accompanied with peaks of the relative expression of every molecule examined within the time range, albeit to varied extents (Table 3, data of 14 dpi). Notably, the temporal pattern expression of *RORγt*, *CCR6*, and other Th17-related molecules suggested the progression of Th17 cells from secondary lymphoid organs to the retinas during the initiation and effector phases of disease (data of 7 and 14 dpi, Table 1 and Table 3). In addition, decreased expression of the subunit p40 of *IL-23* was observed at 7 dpi of both draining lymph nodes (Table 1) and the retinas (Table 3), while significantly unbalanced expression was observed between *IL-23p40* and *IL-23p19*, with the p40 induced in excess over the other subunit at the retinas for the time points after 14 dpi (Table 3). Moreover, the relative expressions of *CD4* and *CD8* were increased to 36.13 fold versus 9.58 fold of the individual control, with the level of *CD4* expression significantly higher than that of *CD8*, in addition to the 5,202.54 fold upregulation of *MHC-II* and 8.63 fold increase of *MHC-I* expression at the equivalent time point (Table 3, data of 14 dpi). These quantitative PCR data showed that Th17 cells were at least one subset of the major pathogenic effector T cells involved in severe ocular inflammation. This was further supported by data obtained from retina immunochemistry with IL-17 antibody, which showed that intensive IL-17-positive infiltrating immune cells were observed especially at 14 dpi in both the retina and vitreous cavity within the eyes of EAU (Figure 2A,C, arrowheads). In addition, the morphological and regional features of the positive-staining cells resembled cells of lymphoid origin (Figure 2C, arrowhead).

Retinal expression of Treg-related molecules also significantly increased at 14 dpi. These included the transcription factor *Foxp3* (10.59 fold upregulation), chemokine receptor *CXCR5* (8.08 fold upregulation), the surface molecule *CD25* (146.02 fold upregulation), and related molecules, some of which overlapped with Th17-related molecules (i.e., *TGF-β* and *CD4*; Table 3, data of 14 dpi). These results indicated that at the peak of EAU the infiltrating immune cells were highly heterogeneous, while immune balance and regulation were operated by significant induction of Treg cells.

### Resident neural cells and infiltrating microglia are activated during the course of EAU

**Figure 3:**
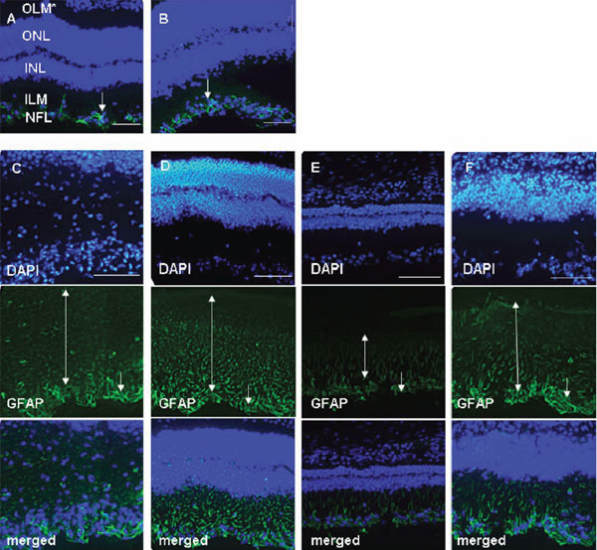
Activation patterns of resident retinal astrocytes and Müller cells during EAU. The antibody against glial fibrillary acidic protein (GFAP; green) was used to identify astrocytes and Müller cells; 2-(4-amidinophenyl)-6-indolecarbamidine dihydrochloride (DAPI; blue) was applied to counterstain the cell nuclei. In the CFA control (**A**) and EAU 7 dpi group (**B**), there were no detectable GFAP in the endfeet of Müller cells (ILM), and GFAP staining was limited within astrocytes (NFL). Panels **C** (14 dpi), **D** (21 dpi), **E** (28 dpi), and **F** (35 dpi) show that GFAP became detectable throughout the Müller cell cytoplasm from INL to ONL (bidirectional arrows). In addition, positive staining widened and enlarged in astrocytes (arrows), which turned pleomorphic (hypertrophy of the cell body and nucleus, elongation of cytoplasmic processes, irregular in shape) and continuously activated from 14 to 35 dpi. Data represent at least three repeated experiments. OLM, outer limiting membrane; ONL, outer nuclear layer; INL, inner nuclear layer; ILM, inner limiting membrane; NFL, nerve fiber layer. The size bar represents 200 μm (10×).

**Figure 4:**
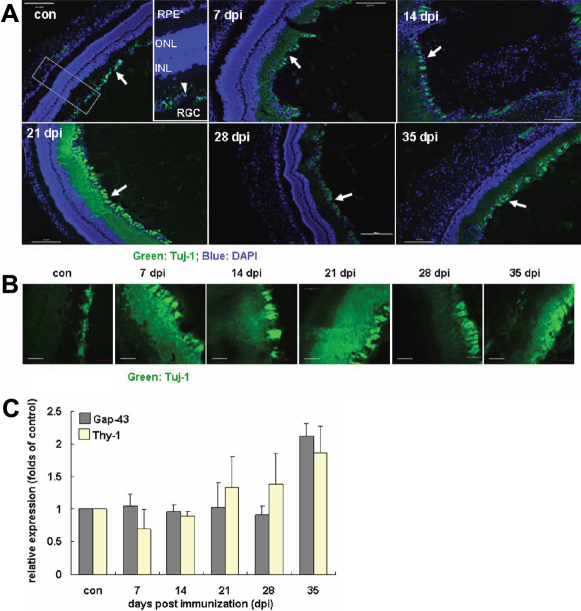
Status of the RGC during EAU. Tuj-1 staining (green) by an antibody against neuronal β3-tubulin in cell bodies and axons showed microtubule stability of the neuronal cells. DAPI (blue) was applied to counterstain the cell nuclei. **A**: During the course of EAU, Tuj-1*-*positive staining increased and spread at 7 dpi, appeared disrupted at the peak of inflammation at 14 dpi, and became the most intensive at 21 dpi with the restoration of normal retinal architecture. The staining was then downregulated at 28 dpi but became relatively stronger again at 35 dpi. The size bar represents 200 μm (10×). **B**: High magnification observation (40×) on the Tuj-1-positive staining showed that, while microtubules of the neuronal axons were destabilized and disassembled when the disease progressed, they gradually restored their features at later stages of EAU. Compared to the staining of the control and that at 28 dpi, distribution of Tuj-1-staining at 35 dpi indicated that the neuronal activities underwent waves of adjustment at the later phase of disease. **C**: Relative expression of thymocyte differentiation antigen 1 (Thy-1) and growth-associated protein-43 (Gap-43) during EAU by real-time RT–PCR. Data represent at least three repeated experiments. RPE, retinal pigment epithelium; ONL, outer nuclear layer; INL, inner nuclear layer; RGC, retina ganglion cells. The size bar represents 50 μm (40×).

To further observe the response of resident retinal neural cells during the course of disease, glial fibrillary acidic protein (GFAP)-positive astrocytes and neuron-specific class III β-tubulin (Tuj-1)-positive RGC were analyzed by fluorescent microscopy. Increased GFAP expression represented activated astrocytes, Müller cells, and gliosis of neurodegeneration [23]. In the CFA control (Figure 3A) and EAU 7 dpi (Figure 2B and Figure 3B) groups, there was no detectable GFAP in Müller cells and the labeling only occurred in astrocytes. At 14, 21, 28, and 35 dpi (Figure 3C-F), GFAP was detectable throughout the Müller cell cytoplasm and GFAP^+^ astrocytes became wider and significantly pleomorphic with their processes enlarged. Such morphological changes showed significant activation of the retinal astrocytes. In parallel, Tuj-1*-*positive staining increased and spread from 7 dpi, became the most intensive at 21 dpi, and was then slowly downregulated (Figure 4A,B). To verify RGC loss or axonal regeneration during the course of EAU, we measured the relative expression of thymocyte differentiation antigen 1 (*Thy-1*) and growth-associated protein-43 (*Gap-43*) by real-time PCR. *Thy-1* is a marker specific for RGC, and reduced expression of *Thy-1* at the mRNA level was reported to be highly relevant to the state of RGC [24]; whereas as a neuron-specific protein, Gap-43 is considered a crucial component of the axon and presynaptic terminal [25]. Data indicated that, although the expression levels of these two factors were equivalent at each stage of disease, the expression of both factors was increased to some extent by 1.87 fold (*Thy-1*) and 2.12 fold (*Gap-43*) at 35 dpi, compared to the control (Figure 4C). Collectively, this expression pattern of *Thy-1* and *Gap43* at the later stage of EAU (Figure 4C) was consistent with the sets of fluorescent microscopy data in neural cells in which the increased again expression of GFAP- and Tuj-1 staining was found at 35 dpi (Figure 3, Figure 4A,B); and the set of real-time PCR data on the expression of T-cell-related molecules at the same time (Table 3).

**Figure 5:**
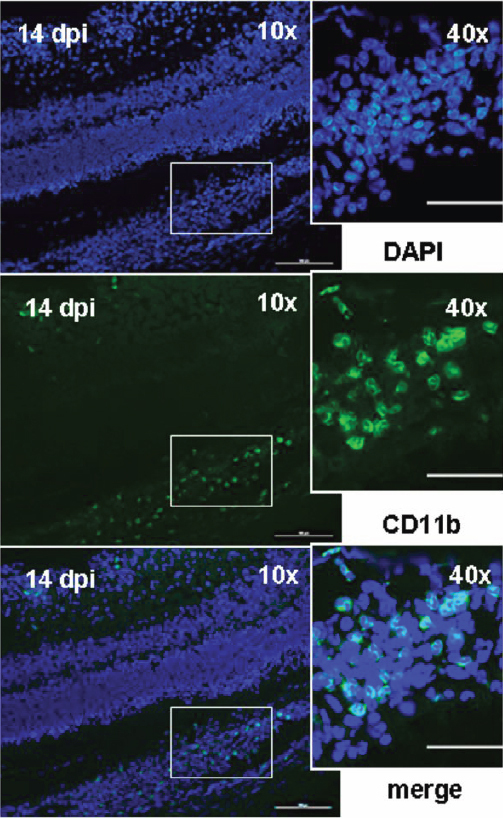
Infiltrating macrophages or microglia were involved in the peak of disease at 14 dpi. CD11b (green) was stained as the β-integrin marker of microglia or mature macrophages. DAPI (blue) was applied to counterstain the cell nuclei. To determine the involvement of infiltrating or resident microglia, CD11b expression was examined in the retina during the course of EAU. Detectable CD11b-positive staining (green) was only observed at 14 dpi but not at other stages of disease (data not shown). The positive staining indicated that CD11b was a cell-surface molecule (CD11b, middle column 40×). The size bar (left panels) represents 200 μm (10×) and the size bar (right panels) represents 50 μm (40×).

To determine if infiltrating or resident microglia were involved in the course of EAU, we also examined *CD11b* expression in the retina. We found a significant number of *CD11b*+ cells at 14 dpi (Figure 5) but not at other stages of EAU (data not shown). This showed that, as infiltrating immune cells, microglia might be mainly involved in the peak of disease, especially when intraocular inflammation is intensive.

### Pattern expression of the overlapping molecules indicated their role in promoting resolution of the monophasic EAU

Although the previous results demonstrated Th17 cells as a major pathogenic factor responsible for the peak of disease, retinal expression of most T-cell-related factors did not return to that of their respective control at a later phase of disease (Table 3). Starting from 21 dpi, the stage of EAU was defined as the resolution stage during which the retina underwent significant clinical recovery (Figure 1A). A similar expression pattern in which the expression was reduced from the peak of EAU to 21 and 28 dpi but then increased again at 35 dpi was observed for *IL-17*, *IL-6*, *TGF-β*, *IL-23p40*, *RORγt*, *MHC-II*, *MHC-I*, and *Foxp3*. In addition, expression of *IL-17R*, *CD4*, *CCR6*, *CXCR5*, and *CD25* was significantly increased as compared to each of the controls at this phase, although the level of expression was far less than that of the respective molecules at 14 dpi (Table 3). Significant correlation was found between the kinetic expression of *IL-17* and the set of individual data of most of these molecules in EAU retinas (p<0.05 or 0.01, Table 3). In contrast, most of the molecules only slightly changed in the peripheral lymphoid organs of EAU at the same period of time, with the exception of *IL-17*, whose expression increased up to 3.21 fold at 28 dpi and remained increased by 1.94 fold at 35 dpi compared to the control (Table 1). This might specify functional properties of these factors in the local environment. Based on these data we hypothesized that these immune-based factors might also be produced by resident neural cells and function as factors to promote retinal structure reconstruction and function.

Immunohistochemistry with an IL-17-specific antibody supported our hypothesis, at least to some extent. Consistently high levels of IL-17 expression were observed in the retina of EAU where RGC and astrocytes resided from 14 dpi compared to that of the controls. Closer observation of IL-17-positive staining cells demonstrated the intracellular staining and that morphology of these cells resembled astrocytes. However, other than the location, we have no further evidence to prove that the IL-17-positive staining cells were indeed RGCs. These results suggested that kinetic expression of IL-17 by resident neural cells may indicate a process of resolution in EAU.

## Discussion

The present study indicated how well organized T-cell activity, measured by relative expression of multiple T-cell-related factors at the mRNA level, synchronized with the initiation of the autoimmune inflammation and thereafter resolution of a monophasic EAU with Lewis rats. The immune balance was achieved several times, one at the peak of disease (14 dpi) and another at the resolution phase (35 dpi) during the recorded observation period, through the coordinated expression of Th17- and that of Treg-associated molecules. In addition, as these T-cell-related molecules are not functionally exclusive to immune cells, the expression pattern of these factors and immunochemistry with an IL-17 antibody indicated that there may be intensive crosstalk between the infiltrating immune cells and resident neural cells, which were significantly activated during the course of disease.

Our data on kinetic expression of multiple T-cell-related factors in vivo indicate novel findings that partial states of differentiation or T-cell flexibility, in which components of Th17 and Treg phenotypes were co-expressed, may dominate effector/regulatory or even memory CD4 cell populations during the course of autoimmune inflammation. In vitro studies indicated that significant number of CD4 T cells co-expressing Foxp3 and ROR-γt were found in human peripheral tissues; these cells produced IL-17 and powerfully inhibited the proliferation of CD4 responder cells ex vivo [19]. The existence of IFN-γ^+^IL-4^+^ cells under Th2 culture conditions implies that partially differentiated Th1 cells retain their capability to become IL-4-producing cells [26]. After three to four rounds of in vitro Th2 priming, most fully differentiated Th2 cells failed to generate IFN-γ, which correlates with the failure of T-bet (T box expressed in T cells) induction in these cells [27]. These demonstrate that the plasticity of T cells may depend on their differentiation state. However, substantial evidence suggests that the in vivo effector/regulatory CD4 T cells may have undergone pathways of differentiation that vary from those established in vitro [28]. As the features of T-cell heterogeneity and plasticity are highly inter-related, the complexity involved in T-cell-mediated autoimmune inflammation in vivo cannot be well understood by activities of only a few key molecules of T cells. Our optimized real-time PCR measurement on parallel activities of multiple interconnected molecules, related to two subsets of T cells at the mRNA level, is designed adaptively, and the data reflect a certain part of T-cell biology in vivo during a period of time when an autoimmune inflammation was initiated and resolved.

The same sets of our data further elucidate that immune balance and regulation is well achieved by relevant activities of pro-inflammatory Th17 cells and immunosuppressive Treg at certain key points during the course of disease. These activities were reflected by coordinated expression of inter-related signature cytokines, master regulator of transcriptional factors, and key cell surface molecules that belong to either of the two T-cell lineages, although some of the molecules functionally overlapped. Overall, the dosage and timing of T-cell activities are well correlated with disease progression and resolution. However, other molecules that are key to Th1 and Th2 subsets should also be tested for measurement on more integrated T-cell activities involved in autoimmune inflammation in vivo. Such PCR measurements should also be applied in recurrent EAU. Thus, the varied T-cell biology between monophasic and recurrent autoimmune disease models may elucidate in detail the role of T-cell-mediated immune responses in disease pathology and help to develop treatment strategies. Nevertheless, heterogeneity and plasticity of T cells may be far more complex. For example, high levels of master regulator gene expression are usually correlated with the phenotype of the proper Th subsets. Some effector functions of T cells may not require these factors to be highly expressed [29]. The functionality of the factor may depend on the cell milieu or, more specifically, on the relative amounts of other vital transcription factors in a kinetic manner. Moreover, further measurements on key molecules at the protein level and epigenetic regulation at the DNA level are also necessary to help understand this complexity [30].

During the course of an active autoimmune inflammation, the infiltrating immune cells are actively interacted with the local neural tissues; thus these immune cell-related molecules may not be exclusive to any cell type or individual system [31] but function as mediators. In our results, the most upregulated expression of MHC-II at the EAU retina indicated that this molecule may be actively involved in antigen presentation to CD4 T cells by resident neural tissue during the course of EAU. In addition, we showed that IL-17 was also produced by retinal astrocytes or RGCs, especially at the later phase of EAU. As a pro-inflammatory cytokine, IL-17 was also shown to have pleiotropic and environment-specific protective functions [8,9]. An earlier structure study revealed significant structural similarity between IL-17 family members and neurotrophins [32]. To further support this notion, significant correlation (p<0.05 or 0.01) was found between the series data of expression of *IL-17* and that of *IL-6*, *TGF-β*, *IL-23p40*, *CD4*, and *MHC-II* in EAU retinas. Interestingly, some of these factors have also been shown to have potent functions, for example, neurotrophins, such as IL-6 [33] and TGF-β [34]. Furthermore, co-culture with activated CD4 T cells resulted in the destabilization of microtubules detected by the Tuj-1 antibody against β3-tubulin in neuronal axon [35]. From our results, significant reorganization of β3-tubulin in RGCs at 35 dpi of retina of EAU animals closely correlated with increased expression of those immune-based molecules at the same time compared to individual features at 28 dpi and the controls. Such parallel data strongly suggest that these immune-based molecules influence neuronal activities and functions at the local organ during the disease.

In conclusion, based on the organ-specific autoimmune model of monophasic EAU, our data provide novel findings regarding in vivo mechanisms of dynamic effector/regulatory T-cell subsets on immune regulations and functional interactions between the infiltrating immune cells and resident neural cells mediated by T cell-related factors during the course of disease.
